# Custom selected reference genes outperform pre-defined reference genes in transcriptomic analysis

**DOI:** 10.1186/s12864-019-6426-2

**Published:** 2020-01-10

**Authors:** Karen Cristine Gonçalves dos Santos, Isabel Desgagné-Penix, Hugo Germain

**Affiliations:** 1 Department of Chemistry, Biochemistry and Physics, Université du Québec à Trois-Rivières, Trois-Rivières, QC G9A 5H7 Canada; 2 Plant Biology Research Group, Université du Québec à Trois-Rivières, Trois-Rivières, QC G9A 5H7 Canada

**Keywords:** Next-generation sequencing, Housekeeping genes for qPCR, R script

## Abstract

**Background:**

RNA sequencing allows the measuring of gene expression at a resolution unmet by expression arrays or RT-qPCR. It is however necessary to normalize sequencing data by library size, transcript size and composition, among other factors, before comparing expression levels. The use of internal control genes or spike-ins is advocated in the literature for scaling read counts, but the methods for choosing reference genes are mostly targeted at RT-qPCR studies and require a set of pre-selected candidate controls or pre-selected target genes.

**Results:**

Here, we report an R-based pipeline to select internal control genes based solely on read counts and gene sizes. This novel method first normalizes the read counts to Transcripts per Million (TPM) and then excludes weakly expressed genes using the DAFS script to calculate the cut-off. It then selects as references the genes with lowest TPM coefficient of variation. We used this method to pick custom reference genes for the differential expression analysis of three transcriptome sets from transgenic *Arabidopsis* plants expressing heterologous fungal effector proteins tagged with GFP (using GFP alone as the control). The custom reference genes showed lower coefficient of variation and fold change as well as a broader range of expression levels than commonly used reference genes. When analyzed with NormFinder, both typical and custom reference genes were considered suitable internal controls, but the custom selected genes were more stably expressed. geNorm produced a similar result in which most custom selected genes ranked higher (i.e. were more stably expressed) than commonly used reference genes.

**Conclusions:**

The proposed method is innovative, rapid and simple. Since it does not depend on genome annotation, it can be used with any organism, and does not require pre-selected reference candidates or target genes that are not always available.

## Background

RNAseq is a technique used since the pioneer studies of R Lister, RC O’Malley, J Tonti-Filippini, BD Gregory, CC Berry, AH Millar and JR Ecker [1] (*Arabidopsis thaliana*), U Nagalakshmi, Z Wang, K Waern, C Shou, D Raha, M Gerstein and M Snyder [2] (*Saccharomyces cerevisiae*), BT Wilhelm, S Marguerat, S Watt, F Schubert, V Wood, I Goodhead, CJ Penkett, J Rogers and J Bähler [3] (*Schizosaccharomyces pombe*), and A Mortazavi, BA Williams, K McCue, L Schaeffer and B Wold [4] (*Mus musculus*). This technique allows the combination of transcript discovery and expression level quantification in a single assay and has an unlimited dynamic range of detection compared to microarray or RT-qPCR [5, 6].

For differential expression studies, the gene expression values must be comparable between samples, which means that count data should be normalized for sequencing depth and other biases such as transcript length, GC content and transcript coverage. Reads/Fragments per Kilobase per Million (RPKM or FPKM) and Transcripts per Million (TPM) both normalize count data by transcript length and sequencing depth [7], but they may give biased results in the presence of highly expressed genes or when a lot of the genes are expressed in only one sample [8]. This is because one differentially expressed gene shifts the sequencing effort distributed to the others and all genes appear to be differentially expressed [9–11]. Other methods such as relative log expression (DESeq2) and trimmed mean of M-values (edgeR) can work with the carry-over effect of highly expressed genes [10].

The comparison of different softwares for RNAseq analysis is a recurrent subject in the literature [12–14] and many authors argue over the benefits of using housekeeping genes or spike-in controls to scale the count data, yet the evaluation of the reference genes used for RNAseq data analysis is not as common. When using internal or external control genes, the normalization is first performed on the controls and the result is used to normalize the other genes. The use of external spike-ins is advocated for introducing little error into the read counts, allowing identification of global shifts in gene expression [15–17]. However, reports have shown mixed performances with different normalization methods [18], resulting in high false discovery rates and false positive rates [19]. These may show differences in amplification depending on the type of tissue studied or the protocol for mRNA enrichment [20].

One alternative for external spike-ins is the use of internal control genes, as it is done in qPCR studies. Typical control genes are actin, tubulin, elongation factor 1, polyubiquitin and ribosomal RNAs, though the stability of expression of several of those is dependent on the conditions studied [21]. To solve this issue, different algorithms were proposed to find stably expressed genes, mostly for qPCR applications, but they need a set of predefined genes of interest (RefGenes, T Hruz, O Laule, G Szabo, F Wessendorp, S Bleuler, L Oertle, P Widmayer, W Gruissem and P Zimmermann [22]) or a set of pre-selected candidate reference genes (geNorm, J Vandesompele, K De Preter, F Pattyn, B Poppe, N Van Roy, A De Paepe and F Speleman [23]; NormFinder, CL Andersen, J Ledet-Jensen and T Ørntoft [24]; BestKeeper, MW Pfaffl, A Tichopad, C Prgomet and TP Neuvians [25]). The most frequent approach is to take previously identified stably expressed genes, as done by B Zhuo, S Emerson, JH Chang and Y Di [11], this however does not ensure that the selected genes will show stable expression in the studied organism and conditions.

Here we propose a simple and fast method to identify the most stably expressed genes for each experimental condition. Our method is aimed at differential expression studies and represents a simple way to select custom reference genes for any species or any type of experiments, so they can be used in the normalization step of differential expression analysis algorithms, and does not necessitate spike-ins. It alleviates the problem inherent to predefined reference genes, which may not be stably expressed across experimental set-ups and are applicable to a single species.

## Results

**Fig. 1:**
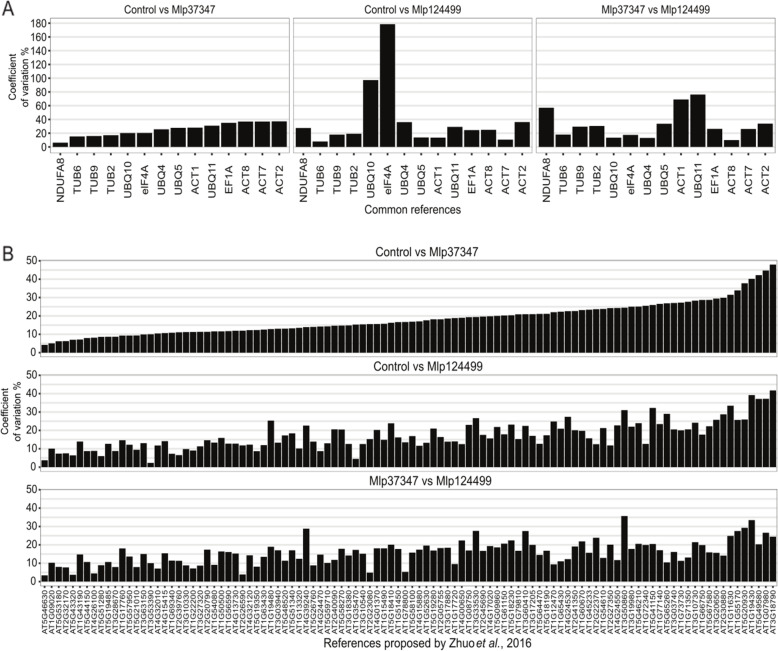
Evaluation of coefficient of variation distribution in the three transcriptome data sets. **a** among a set of 14 commonly used reference genes and **b** a set of 104 reference genes proposed by B Zhuo, S Emerson, JH Chang and Y Di [11]

Initially three RNAseq transcriptomes were generated using *Arabidopsis* transgenic plants expressing GFP alone (control) or GFP-fused to fungal effector genes (*Mlp37347* and *Mlp124499*). We tested the normalization of our RNAseq data using two sets of reference genes: commonly used reference genes (Table 1) and the 104 stably expressed *Arabidopsis* genes proposed by B Zhuo, S Emerson, JH Chang and Y Di [11]. The first set of reference genes was assessed for stability in three different permutations of the transcriptome sets as shown in Fig. 1a (panel 1: Mlp37347 vs Control, panel 2: Mlp124499 vs Control, panel 3: Mlp124499 vs Mlp37347). In each case, high levels of coefficient of variation, ranging from 4.9% (NDUFA8 in Mlp124499 vs Mlp37347) to 41.5% (tubulin 6 in Mlp124499 vs Mlp37347) were obtained. Next, we performed the same analysis using the 104 genes proposed by B Zhuo, S Emerson, JH Chang and Y Di [11]. For the three permutations of the transcriptome sets, important fluctuations in the coefficient of variation were observed ranging from 2.9 to 49% (Fig. 1b). Finally, we did the same for the set of 30 genes selected by T Czechowski, M Stitt, T Altmann, MK Udvardi and W-R Scheible [26] for several plant tissues (Additional file 1). These results demonstrate that neither the commonly used reference genes, nor the 104 reference genes proposed by B Zhuo, S Emerson, JH Chang and Y Di [11] were stably expressed in our conditions.

**Table 1:** Common reference genes used in this study for comparison against custom selected reference genes

Symbol	Name	ATG
Actin 2	ACT2	AT3G18780
Actin 7	ACT7	AT5G09810
Actin 8	ACT8	AT1G49240
Adenine phosphoribosyltransferase 1	APT1	AT1G27450
Elongation factor 1-α	EF1α	AT5G60390
Eukaryotic translation initiation factor 4A-1	elF4A	AT3G13920
NADH-ubiquinone oxidoreductase 19-kDa subunit	NDUFA8	AT5G18800
Tubulin β-2/β-3 chain	TUB2	AT5G62690
β-tubulin 6	TUB6	AT5G12250
Tubulin β-9 chain	TUB9	AT4G20890
Polyubiquitin	UBQ4	AT5G20620
Ubiquitin extension protein	UBQ5	AT3G62250
Polyubiquitin	UBQ10	AT4G05320
Polyubiquitin	UBQ11	AT4G05050

In order to search for more stably expressed genes, we developed a custom method to select reference genes using only one’s own RNAseq data. We first used a R function to transform the count data into Transcripts per Million [27] and calculate the average TPM and coefficient of variation for each gene. We then used the DAFS function [28] to calculate a cut-off for the exclusion of weakly expressed genes. Finally, the 0.5% remaining genes with lowest coefficient of variation were selected as reference genes (R-package “CustomSelection” [29]). This pipeline is thereafter referred to as the custom selection script.

**Fig. 2:**
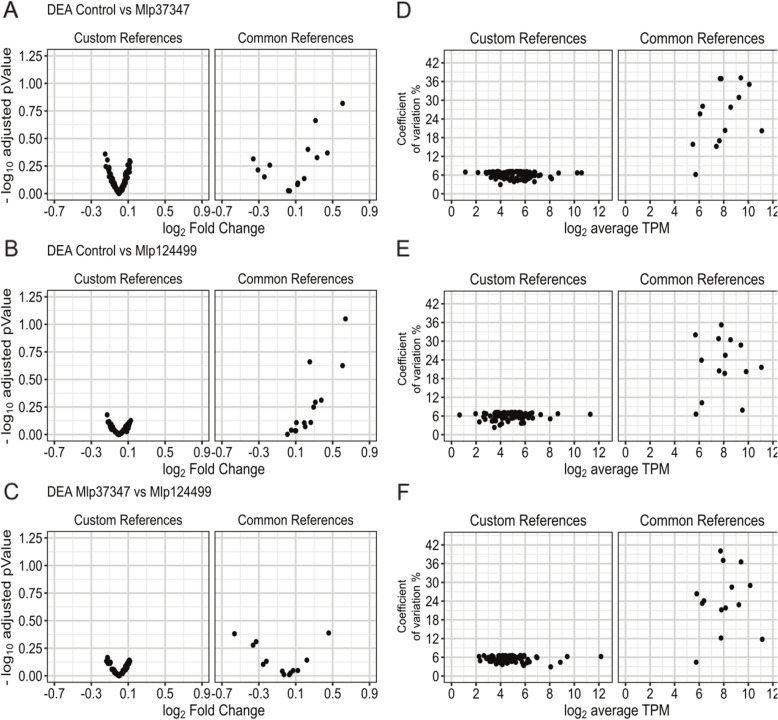
Comparison the four sets of reference genes in relation to coefficient of variation level and log_2_ TPM for **a** Mlp37347 vs Control, **b** Mlp124499 vs Control and **c**) Mlp124499 vs Mlp37347

**Fig. 3:**
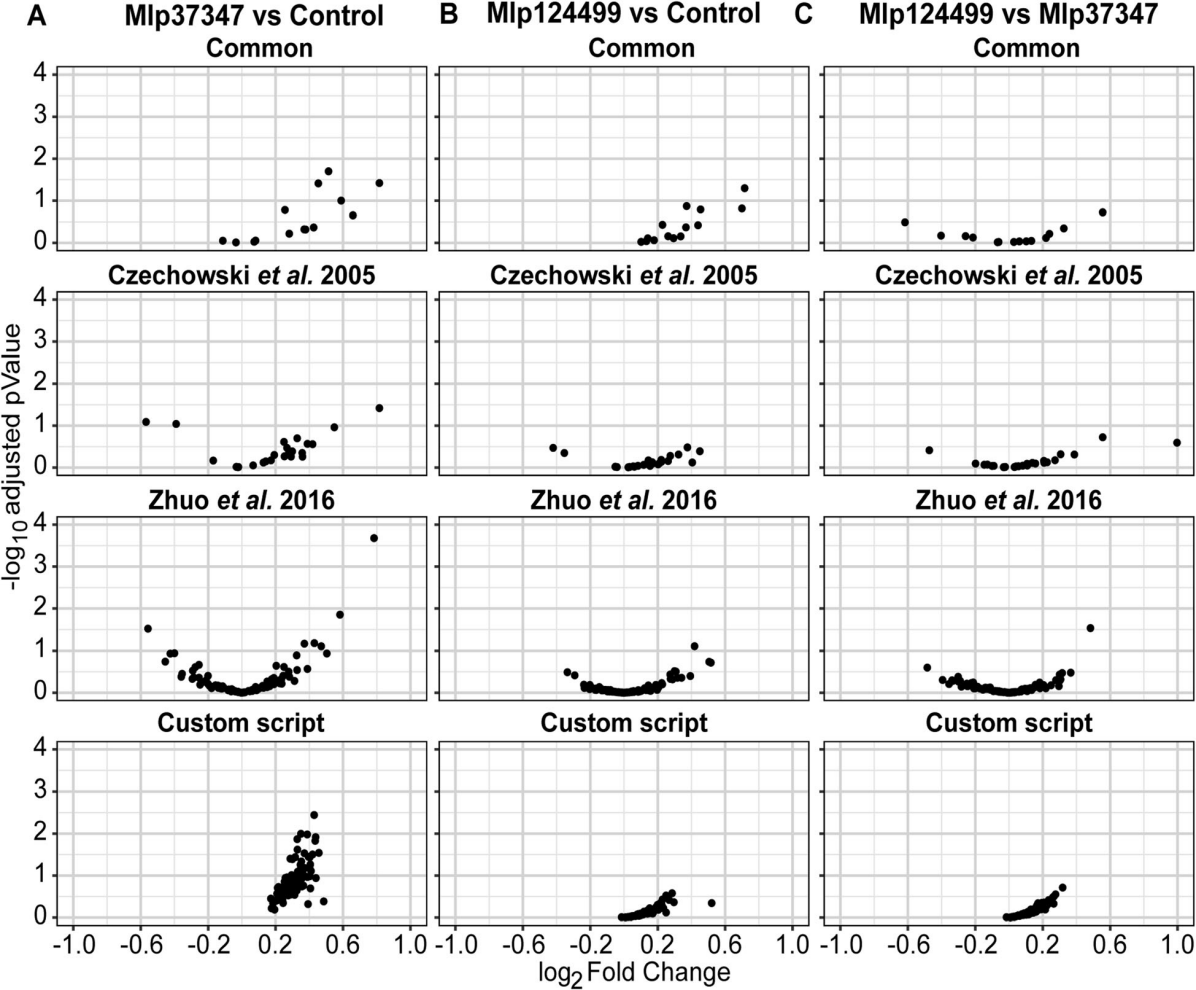
Comparison of the four sets of reference genes in relation the distribution of log_2_ fold Change by -log_10_ adjusted *p*-value for **a** Mlp37347 vs Control, **b** Mlp124499 vs Control and **c** Mlp124499 vs Mlp37347

To test the developed method, we used the same transcriptome sets described in Fig. 1 (the list of selected genes for each analysis is available in Table 1, Additional file 2). For each transcriptome set, we show in Fig. 2 the average expressing in log_2_ TPM and coefficient of variation of the common reference genes (Common), the set of 30 genes from T Czechowski, M Stitt, T Altmann, MK Udvardi and W-R Scheible [26] (Czechowski et al. 2005), the set of 104 genes from B Zhuo, S Emerson, JH Chang and Y Di [11] (Zhuo et al. 2016) and the genes selected using the CustomSelection package [29] (Custom script). In all pairings the custom selected reference genes show broader range of expression levels and lower coefficient of variation (Fig. 2) than the other sets. Next, we performed a differential expression analysis with DESeq2 [30] without control genes. We show in Fig. 3 the log_2_-transformed fold change by the –log_10_-transformed adjusted *p*-value for each gene set. We can see that the set of genes selected with the custom script shows lower fold change in all cases. We also compared the results of DESeq2 using no reference gene or the four sets indicated above for each permutation. As is shown in Table 2, in all the permutations the analysis without the use of references gives higher number of up-regulated genes than the analyses that use any of the reference sets while resulting in a lower number of down-regulated genes, possibly indicating a shift to downregulation that is not detected without reference genes.

**Fig. 4:**
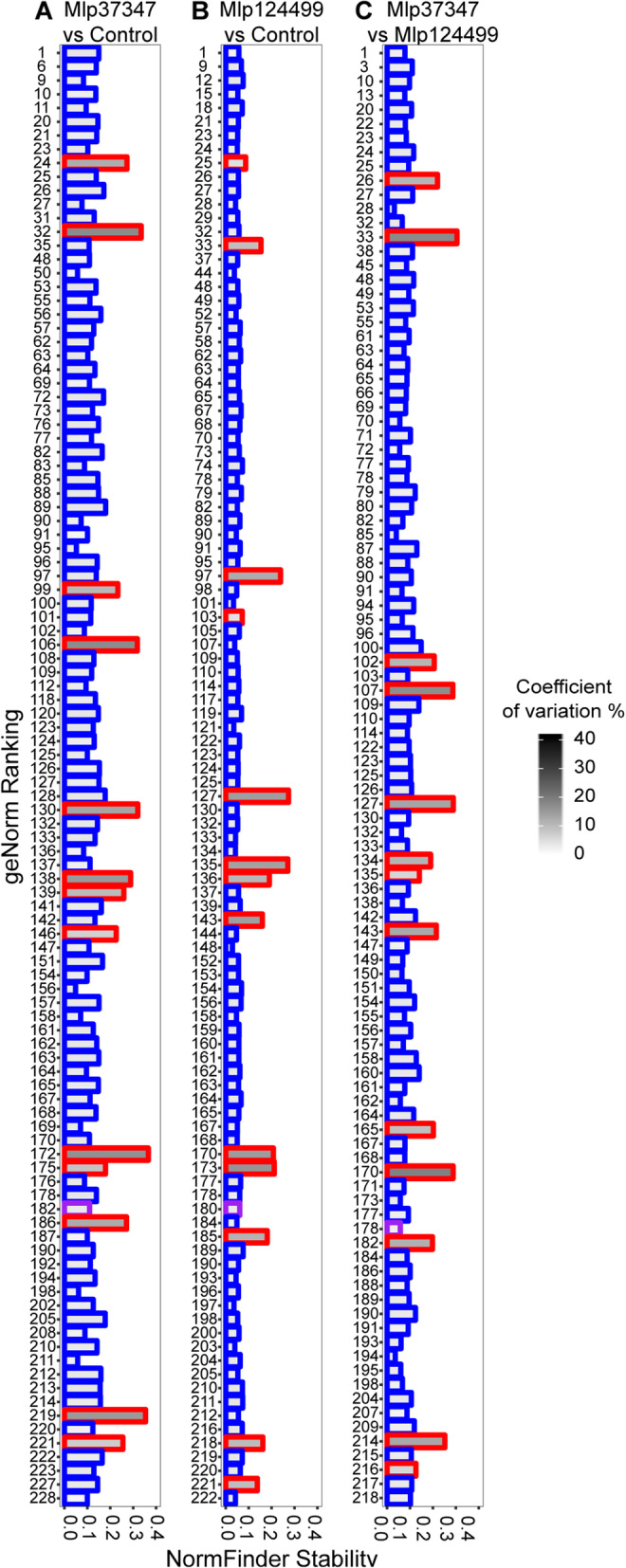
Comparison of custom selected reference genes (blue border) and commonly used reference genes (red border) with geNorm ranking, NormFinder stability index and coefficient of variation for **a** Mlp37347 vs Control, **b** Mlp124499 vs Control and **c** Mlp124499 vs Mlp37347. The bar with purple border indicates the gene (NDUFA8) selected with the custom script that is also present in the common references

To further test the stability of the custom reference genes in our experiment, we used NormFinder [24] and geNorm [23] to compare the four sets of reference genes using log_2_ transformed TPM values. The complete result is presented in the Tables S3-S5 of the Additional file 2. We present in Fig. 4 the comparison of the set of common reference genes against the custom selected reference genes. The gene AT5G18800 (NDUFA8) which is in the set of common references was selected by the custom script in all three permutations and is shown with a purple border. Both sets of genes (custom and common refences) were under the stability threshold of NormFinder (0.5), meaning that the software considers them suitable references genes, however the custom selected genes (shown with a blue border) were more stable than the commonly used genes (shown in red, Fig. 4). This was also the case for most genes tested with geNorm.

## Discussion

The use of reference genes in RNAseq studies is suggested in the literature [15–17], yet the methods for the selection of these genes are designed for qPCR data and require a set of pre-selected reference or target genes or the selection of conditions similar to that of one’s own experiment [22–25], which are not always available. As there is no previous transcriptomic study of plants constitutively expressing fungal effectors and since the information available on these effectors is scarce [31], it is not possible to know a priori their function and which host genes are impacted by the presence of these fungal proteins. For these reasons, we propose a new R-package which enables the selection of custom reference genes regardless of the organisms used or of the experimental conditions.

The method developed here only requires information available from the RNAseq analyses. It uses Transcripts per Million [27] as a proxy for the expression level and the DAFS algorithm [28] to exclude genes with low counts, which may be inactive [32]. We first assessed whether the most commonly used reference genes (Table 1) or two sets of published reference genes for *Arabidopsis* [11, 26] were indeed stably expressed in our experimental conditions. As demonstrated in Fig. 1 and Additional file 1, three sets of reference genes show a high level of coefficient of variation in our experimental conditions, indicating that they were not suitable reference genes for our differential expression analysis.

Having a high level of variability in the expression of the reference genes results in skewed quantitative analysis and may cause the loss of some differentially expressed genes which show modest variation in gene expression [21]. In relation to the reference gene sets, there is minimal overlap between sets published and the ones selected in this article (maximum of 5 genes shared between our set and the set of B Zhuo, S Emerson, JH Chang and Y Di [11] and 2 genes shared between our set and the set of T Czechowski, M Stitt, T Altmann, MK Udvardi and W-R Scheible [26], shown in Additional file 2 Table S3, S4, S5 column J). However, there is extensive overlap in the deregulated genes (up- and down-regulated as shown in Additional file 2: Table S2). This fact demonstrates that all three sets perform well in detecting deregulated genes, however having a references gene set with lower co-variance results in the finding of more de-regulated genes (Additional file 2: Table S2 downregulated) since more subtle deregulation can be detected.

Thus, to alleviate the bias inherent to the use of inappropriate reference genes, we devised a R-based pipeline to select custom reference genes for one’s own experimental data. As presented in Figs. 2 and 3, in all the pairings of the data used, the custom selected reference genes outperformed the other sets of reference genes in their expression stability, presenting lower fold changes and lower coefficient of variation. Our method allows the selection of genes more stably expressed and the selection of more genes as references (the final number is user defined, with the default setting being 0.5% of the expressed genes), giving more reference points, hence more robustness, to the normalization of genes expressed at different levels. The advantage of having a user-defined threshold is that when there is extensive variation in the data, a stringent threshold may result in the selection of few or no genes as references. On the contrary, extremely homogenous data would result in a very large reference gene set, for this reason a user-defined threshold is preferable.

## Conclusions

Our results show the need for a new R-based pipeline for the selection of custom reference genes in transcriptomic studies. Our method can be applied to any organism and to any type of experimental conditions, and can easily be implemented or modified in R. This tool provides an alternative to spike-in controls and represents an improvement over pre-defined reference genes which may not be stably expressed in one’s own experimental conditions.

## Methods

Initial *Arabidopsis thaliana* Columbia-0 were obtained from *Arabidopsis* Biological Resources Center (ABRC). *Arabidopsis* transgenic plants expressing GFP alone (Control) or fused to a candidate secreted effector protein of the fungus *Melampsora larici-populina* (Mlp37347 or Mlp124499), obtained in our laboratory [31], were used for the transcriptome analysis.

RNA was extracted from pooled aerial tissue of 2-week-old soil-grown plants, doing four replicates per genotype, with the Plant Total RNA Mini Kit (Geneaid) using RB buffer following manufacturer’s protocol. The samples were treated with DNAse, then RNA quality was assessed using agarose gel electrophoresis. Libraries were generated with the NeoPrep Library Prep System (Illumina) using the TruSeq Stranded mRNA Library Prep kit (Illumina) and 100 ng of total RNA following manufacturer’s recommendations. The libraries were then sequenced with Illumina HiSeq 4000 Sequencer paired-end reads of 100 nt.

Libraries were trimmed using Trimmomatic [33] (LEADING:4 TRAILING:4 SLIDINGWINDOW:4:20 MINLEN:20) and then the surviving paired reads were aligned to the TAIR10 assembly of the genome of *A. thaliana* with TopHat v2.0.14 [34] in Galaxy [35] (default options, with average mate inner distance varying for each replicate (Additional file 2: Table S6) and standard deviation of mate inner distance of 50 base pairs). The general information of the sequencing results and mapping data is presented in Additional file 2: Table S6, the dataset was deposited in NCBI under BioProject PRJNA528094. Further analyses were done using R software v.3.2.5. Genomic ranges of *Arabidopsis* transcripts were obtained from Ensembl plants [36] with GenomicFeatures and overlaps of sequencing reads with the transcripts were counted using GenomicAlignments [37], using options for paired-end reads and union mode.

We transformed the counts into TPM [27] and calculated the cutoff for active genes with DAFS [28]. We considered as reference the 0.5% of the active genes with the lowest coefficient of variation (R package “CustomSelection” [29]). Next, we used DESeq2 [38] to confirm that the selected genes were not deregulated. Finally, we used geNorm [23] and NormFinder [24] to compare the custom selected reference genes against three sets of genes (a list of 14 commonly used housekeeping reference genes (Table 1), the reference genes selected by T Czechowski, M Stitt, T Altmann, MK Udvardi and W-R Scheible [26] and the 104 reference genes selected by B Zhuo, S Emerson, JH Chang and Y Di [11]), using TPM values for the expression levels.

Description of the R-package. This package has 4 functions, “Counts_to_tpm” (to convert read counts into TPM values using a named vector with gene lengths) and the read count data frame with the samples as the column names and the genes as row names, “DAFS” (uses the data frame of TPM values, first object of the result from “Counts_to_tpm” to get the threshold for expressed genes), “gene_selection” (uses the data frame of TPM and the result from “DAFS” output a data frame with the selected reference genes, their average TPM and the coefficient of variation of the TPM values) and “customReferences” (calculates internally “Counts_to_tpm”, “DAFS” and “gene_selection” outputs the result from “gene_selection”). The package also includes to datasets for testing: a data frame of counts created with the data used in this article and a named vector with the lengths of genes from Arabidopsis. A Wiki, which is the file README.md of this package, describes a workflow to get the read counts from raw read files.

## Supplementary information

**Additional file 1.** Coefficient of variation level for each of the 30 genes selected by T Czechowski, M Stitt, T Altmann, MK Udvardi and W-R Scheible [26] for each permutation (A: Mlp37347 vs Control; B: Mlp124499 vs Control; C: Mlp124499 vs Mlp37347).

**Additional file 2: Table S1.** TAIR IDs of custom selected references for each transcriptome permutation. **Table S2.** DESeq2 results summary of analysis without reference genes or with different reference sets (Custom selected, from T Czechowski, M Stitt, T Altmann, MK Udvardi and W-R Scheible [26], from B Zhuo, S Emerson, JH Chang and Y Di [11] or Commonly used references). Table presents the number of genes found up- and down-regulated in **Table S3** to **S5.** Summary of the results of several analyses for all the genes evaluated in this article: Column A: TAIR ID; Column B: ranking calculated with geNorm with the function “selectHKs” from the R package “NormqPCR”; Column C: average TPM value; Column D: coefficient of variation of the TPM values; Column E: the difference of expression of a gene between two samples calculated with NormFinder; Column F: the common standard deviation of the expression of a gene between two samples calculated with NormFinder; Column G: stability measure from NormFinder; Column H: log2-transformed fold change of each gene calculated with DESeq2 without using reference genes; Column I: adjusted *p* value of the gene deregulation calculated with DESeq2 without using reference genes; Column J: sources that identified the gene as a reference, when more than one source selected the gene as reference they are separated by a “;”. **Table S3.** Permutation Mlp37347 vs Control; **Table S4.** Permutation Mlp124499 vs Control; **Table S5.** Permutation Mlp124499 vs Mlp37347. **Table S6.** Metadata of samples used; replicate identification, number of sequenced reads, average length of the separation between two paired reads, number of reads after trimming and filtering and number of aligned reads for each of the 4 replicates of the three samples used in this study.

## Data Availability

The dataset used herein was deposited in NCBI-SRA under BioProject PRJNA528094.

## Abbreviations

ABRC: *Arabidopsis* Biological Resources Center
ACT2: Actin 2
ACT7: Actin 7
ACT8: Actin 8
APT1: Adenine phosphoribosyltransferase 1
DAFS: Data-Adaptive Flag Method for RNA-Sequencing Data
EF1α: Elongation factor 1-α
elF4A: Eukaryotic translation initiation factor 4A-1
FPKM: Fragments per Kilobase per Million
GFP: Green Fluorescent Protein
mRNA: messenger Ribonucleic Acid
NDUFA8: Nicotinamide adenine dinucleotide-ubiquinone oxidoreductase 19-kDa subunit
qPCR: quantitative Polymerase Chain Reaction
RNA: Ribonucleic Acid
RNAseq: RNA sequencing
RPKM: Reads per Kilobase per Million
RT-qPCR: Reverse Transcription quantitative Polymerase Chain Reaction
TPM: Transcripts per Million
TUB2: Tubulin β-2/β-3 chain
TUB6: β-Tubulin 6
TUB9: Tubulin β-9 chain
UBQ10: Polyubiquitin
UBQ11: Polyubiquitin
UBQ4: Polyubiquitin
UBQ5: Ubiquitin extension protein
