# Correction to: Custom selected reference genes outperform pre-defined reference genes in transcriptomic analysis

**DOI:** 10.1186/s12864-021-07743-7

**Published:** 2021-08-09

**Authors:** Karen Cristine Gonçalves dos Santos, Isabel Desgagné-Penix, Hugo Germain

**Affiliations:** 1 Department of Chemistry, Biochemistry and Physics, Université du Québec à Trois-Rivières, Trois-Rivières, QC G9A 5H7 Canada; 2 Plant Biology Research Group, Université du Québec à Trois-Rivières, Trois-Rivières, QC G9A 5H7 Canada

**Correction to: BMC Genomics 21, 35 (2020)**

**https://doi.org/10.1186/s12864-019-6426-2**

Following publication of the original article [1], the author reported that although they used the word “covariance” throughout the manuscript, the actual statistics used for selection of reference genes was the “coefficient of variation”.

Note that this does not affect the method, nor the scripts present in GitHub (https://github.com/KarenGoncalves/CustomSelection) to process the datasets.

The original article has been corrected.
