# Supplementary figures and images for: Custom selected reference genes outperform pre-defined reference genes in transcriptomic analysis
Source: BMC Genomics. 2020 Jan 10;21:35. doi: 10.1186/s12864-019-6426-2 (PMC6954607; doi:10.1186/s12864-019-6426-2)

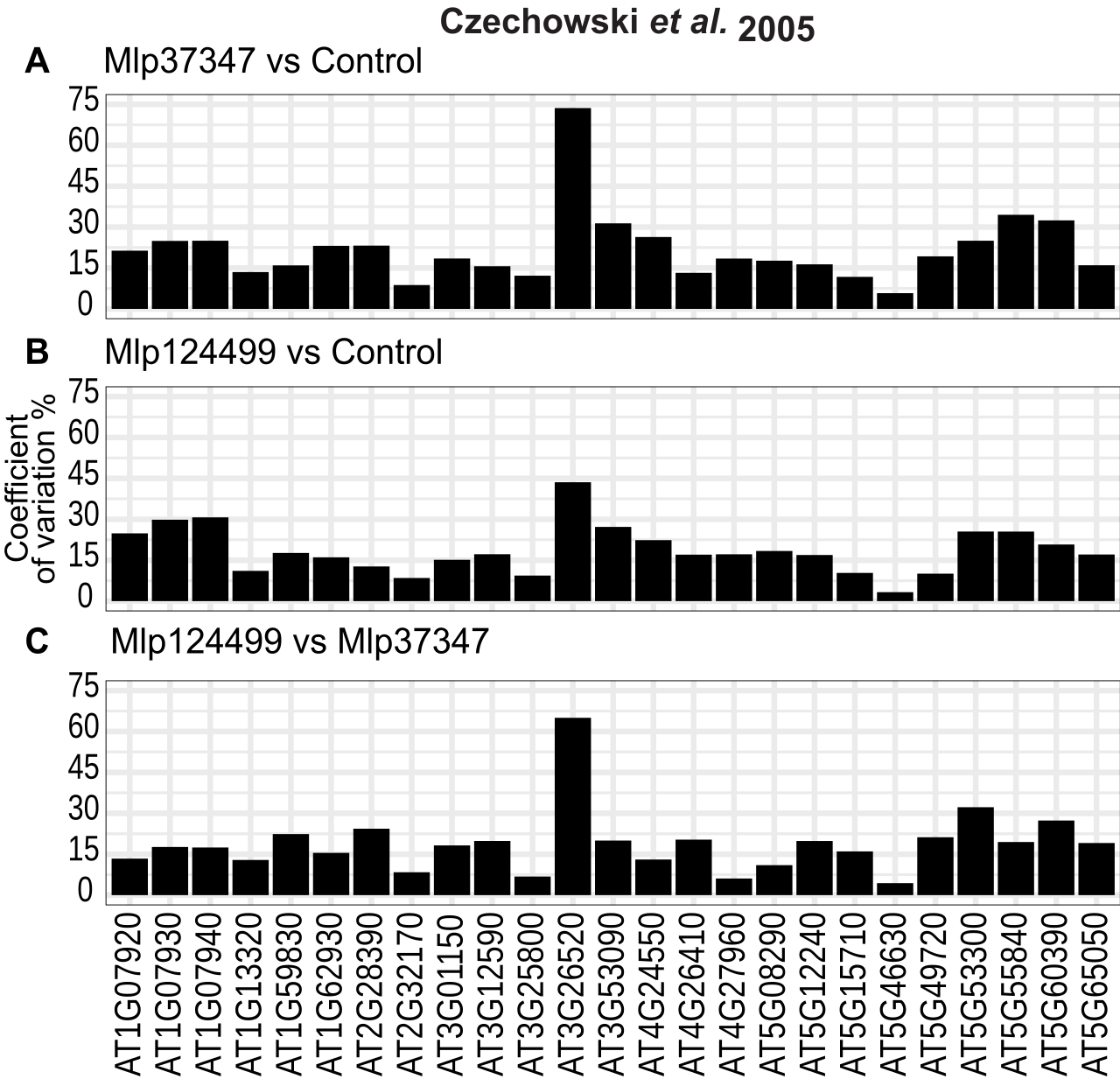

Supplement: Supplementary file 1 — Additional file 1. Coefficient of variation level for each of the 30 genes selected by T Czechowski, M Stitt, T Altmann, MK Udvardi and W-R Scheible [26] for each permutation (A: Mlp37347 vs Control; B: Mlp124499 vs Control; C: Mlp124499 vs Mlp37347). [file 12864_2019_6426_MOESM1_ESM.tif]
